# New insights of propolis nanoformulation and its therapeutic potential in human diseases

**DOI:** 10.5599/admet.2128

**Published:** 2024-01-26

**Authors:** Paula Mariana Kustiawan, Putri Hawa Syaifie, Khalish Arsy Al Khairy Siregar, Delfritama Ibadillah, Etik Mardliyati

**Affiliations:** 1 Faculty of Pharmacy, Universitas Muhammadiyah Kalimantan Timur, Samarinda, East Kalimantan 75124, Indonesia; 2 Center of Excellence Life Sciences, Nano Center Indonesia, South Tangerang 15314, Indonesia; 3 Research Center for Vaccine and Drug, National Research and Innovation Agency (BRIN), Bogor 16911, Indonesia

**Keywords:** nanomaterial, nanopropolis, pharmacological action, bioactive compound

## Abstract

**Background and purpose:**

Scientific research is crucial to develop therapies for various disease severity levels, as modern drugs cause side effects and are difficult to predict. Researchers are exploring herbal alternatives with fewer side effects, particularly propolis, which has been validated through in vitro, in vivo, and clinical studies. This will focus on scientific evidence and its supporting technology for developing new bioactive compounds for chronic diseases. Nanotechnology can improve the delivery and absorption of herbal medicines, which often have poor bioavailability due to their high molecular weight and solubility in water, particularly in oral medicines. This technology can enhance propolis's effects through multi-target therapy and reduce side effects.

**Experimental approach:**

All publications related to each section of this review were discovered using the search engines Google Scholar, Scopus, and Pubmed. This was only available for publication between 2013 and 2023. The selected publications were used as references in this review after being thoroughly studied.

**Key results:**

Evaluation of propolis active compounds, the classification of propolis nano formulations, design concepts, and mechanisms of action of propolis nano formulation. Additionally, the challenges and prospects for how these insights can be translated into clinical benefits are discussed.

**Conclusion:**

In the last ten years, a list of nanoformulation propolis has been reported. This review concludes the difficulties encountered in developing large-scale nanoformulations. To commercialize them, improvements in nano carrier synthesis, standardized evaluation methodology within the framework of strategy process improvement, and Good Manufacturing Practices would be required.

## Introduction

Propolis is a bee product that is soft and sticky and has a distinctive odor [1]. Propolis and its extracts have the potential as promising natural therapeutic agents for degenerative diseases, such as diabetes [2-4], cancer [5-7], liver disease[8-10], cardiovascular disease [11,12], complications of bacterial [13,14] and viral infections [15,16]. The main chemicals in propolis are flavonoids, polyphenols and terpenes [17-19]. The potential pharmacological activity of propolis is very broad for health with improvement of immune system [20] and there has been a lot of research ranging from *in silico*, *in vitro* to clinical studies regarding the effects of propolis on health [21,22]. Apart from that, propolis also has several biological activities, including antioxidant [23], anti-inflammatory [24], antibacterial, antifungal, and antiviral [25]. However, propolis has shortcomings in bioavailability, solubility, absorption speed, and low concentration. The effectiveness of propolis is less than optimal [26].

So far, thus degenerative disease can be cured with modern medicine, but it can have harmful side effects [27]. There are limited therapeutic uses in drug delivery research and development. In fact, it is estimated that 40 % of drugs have been marketed and 90 % of drugs have not undergone pharmacological determination [28]. Nano formulations have been designed to make propolis more soluble to improve its effectiveness in dosage, pharmacokinetics, solubility, sustained drug delivery, and safety. Biodistribution of the drug was improved and decreased toxicity with multidrug resistances. Several propolis nanoformulations have been found and proven to have biological effects such as antidiabetic, hepatoprotective, anticancer and others [29]. Formulation types include solid lipid nanoparticles, liposomes, transferosomes, and polymeric nanoparticles. Nanocarriers can extend the half-life of drugs in therapy, improve pharmacokinetic profiles, and increase patient compliance [30].

Nanotechnology is the updated technology to their chemical and physical structure with higher reactivity and solubility of small objects ranging below 100 nm [31]. The stability of a substance increases due to the protection of oxidizing agents, enzymes or other compounds when its bioactive components are converted into nanostructures [32]. The results are promising and could allow scientists to implement the process more quickly and efficiently, and perhaps with lower risk for consumers. That represents technological advances and developments in their size fields [33]. Nanoparticle products show easier absorption by the body due to their smaller size [34], while propolis nano formulation exhibits superior antibacterial and antifungal activity compared to propolis. [35].

However, the current challenge is the question regarding the safety of applying nanopropolis for oral administration for both short-term and long-term treatment [36]. At the same time, the delivery system for oral administration is linked to the propolis nanoformulation to maximize the synergistic phytochemical agents based on the nano delivery system. This review will provide an overview of propolis and explore how nano propolis formulation methods can enhance its therapeutic effects. At the end of the review, we discuss examples of the challenges of nano propolis formulations and prospects as they have been developed for oral administration.

## Active compounds of propolis

The biological activity of propolis that has been intensively explored, as well as the therapeutic advantages associated with its application in numerous health issues, emphasize the influences of bioactive compounds in propolis. The chemical composition of propolis exhibits significant diversity by different botanical origins, geographical locations, bee species, climatic conditions, and timing of bee’s collection. [37-39]. Commonly, propolis consists of 5 % pollens, 5 % vitamin and other organic substances, 10 % of essential oils, 30 % of wax, as well as 50 % of plant resins or balsams as major components [40,41]. Most pharmacologically active substances are contained in resin or balsam propolis, with polyphenols and flavonoids as important active compounds. The therapeutic properties of propolis are determined by the presence of polyphenols and flavonoids. Total phenolics and total flavonoids, including total flavones, flavonols, flavanones, and dihydroflavonones were used to set specific criteria and standard values for the bioactive compounds of propolis [42]. However, most research focuses on propolis produced by *Apis mellifera*, whereas research into the bioactive compounds of propolis produced by stingless bees is limited [43]. Major phenolic and flavonoids compounds in propolis are quercetin, chrysin, kaempferol, galangin, genistein, myricetin, pinocembrin, pinobanksin, naringin, naringenin, coumarin caffeic acid, gallic acid, ferulic acid, cinnamic acid, saponin, coumaric acid, and caffeic acid phenethyl ester (CAPE) [44]. These compounds are derived from specific plants grown in the region of bee hive. Therefore, propolis showed different biological activity depending on its composition. Table 1 shows recent studies on propolis identification compounds and their biological activity through in vitro or in vivo tests.

**Table 1.** Propolis compounds and their biological activity with extractions, analytical methods and bee types.

Propolis major compound	Extract	Biological activity	Bee type / geographical source	Method	Ref
23-hydroxyisomangiferolic acid, 27-hydroxyisomangiferolic acid, cycloartane- triterpenoids, lanostane- triterpenoid	Ethanolic extract	Anti pancreatic cancer cell, through cytotoxic activity against human pancreatic cancer cells	*Trigona minor* / Vietnam	Preparative TLC and NMR spectroscopy	[45]
Isorhamnetin, sulabiorins A and sulabiroins B	Ethanolic extract	Inhibition of xanthine oxidase	*Tetragonula* aff. *biroi* / Sulasewi, Indonesia	RP-HPLC separations, NMR spectroscopy	[46]
Caffeic acid, ferulic acid, p coumaric acid, cinnamic acid, CAPE, quercetin, kaempferol, galangin, chrysin and pinobanksin	Liquid CO_2_ food grade	Antioxidant activity	*Trigona* sp. / Mataram, Indonesia	HPLC	[47]
[6]-dehydrogingerdione, alpha-tocopherol succinate, adhyperforin, 6-epiangustifolin, deoxypodophyllotoxin, kurarinone, xanthoxyletin	Ethanolic extract	Anti-inflammation activity by reduce the paw volume of inflammation in Sprague Dawley rats caused by carrageenan.	*Tetragonula* sp. / Sulasewi, Indonesia	TLC, LC-MS/MS	[48]
Mangiferonic acid, ambonic acid, cycloartenol, mangiferolic acid, ambolic acid	Ethanolic extract diethyl ether fractination	α-glucosidase inhibitory activity and antioxidant activity	*Tetragonula sapiens* / Indonesia	TLC, HPLC, NMR, GC-MS	[49,50]
Cardol	Ethanolic extract	Anti-cancer activity trough induced apoptotic effect on SW620 human colorectal cancer cell line	*Trigona incisa* / Indonesia	TLC and NMR spectroscopic analysis	[51,52]
Tannic acid, gallic acid, catechin, pyrogallol, naringin. vanillic acid, rutin, benzoic acid, trans-cinnamic acid, quercetin, salicylic acid	Ethanolic extract	Hepatoprotective and ameliorative effects on renal toxic	Bangladesh	HPLC	[53]
Caffeic acid, chlorogenic acid, isochlorogenic acid C, isochlorogenic acid A, kaempferol, caffeic acid phenethyl ester (CAPE), quercetin, artepillin C pinocembrin, myricetin, galangin, apigenin	Ethanolic extract	Antioxidant activity and anti-inflammatory effect by reducing TNF-α, IL-6 and IL-1β.	China and Brazil	HPLC	[54]
Fumaric acid, pyrogallol, caffeic acid, t-ferulic acid, quercetin, isorhamnetin, galangin, chrysin, pinobanksin, pinocembrin, pinostrobin, apigenin, t-cinnamic acid	Ethanolic extract	Anti breast cancer and cytotoxic effects against nonaggressive and aggressive BCCL (MCF-7, SK-BR-3, MDA-MB-23)	Turkey, China and Argentina	LC-MS/MS, HPLC	[55]
dihydrobenzofuran, 2,2-diethynylbut- 2-ene-1,4-diol; 1,4-dihydrophenanthrene; pinostrobin chalcone; galangin flavanone; tectochrysin; 10-hydroxybenzo[j]fluoranthene; naringenin; chrysin	Ethanolic extract, dichloromethane	Anti-bacterial activity against S. aureus, E. coli, P. aeruginosa, and anticancer activity through cytotoxic effect on human prostate cancer cell line (LNCaP) and carcinoma cell line (HN5)	*Apis mellifera* / Ardabil city and Polur, Iran	GC/MS	[56]
2'-hydroxyformononetin, vestitol, butein, dalbergin, 7-hydroxyflavone, pinocembrin	Ethanolic extract	Anti-bacterial activity against helicobacter pylori, staphylococcus aureus and shigella flexneri	*Apis mellifera* and *Trigona* sp./ Nepal	HPLC-DAD-MS/MS	[57]
Propolin C, propolin D, propolis F, propolin G	Ethanolic extract	Anti-bacterial activity against S. aureus, B. subtilis, L. monocytogenes and P. larvae	Taiwanese green propolis	HPLC	[58]
Toluene, pentacosane, 1-heptacosanol, 1-triacontanol, lupenone, lupeol, lupeol acetate	supercritical fluid extraction, with CO_2_ and ethanol as co-solvent	Anti-tumor activity through cytotoxic effect on HCT116 and PC3 tumor cell lines	Brazillian red propolis	GC-MS	[59]
Rutin, CAPE, pinobanksin, quercetin, chrysin, kaempferol, pinocembrin, galangin, p-coumaric acid, benzoic acid	Ethanolic extract	Anti-viral activity by Inhibition viral replication of Covid-19 (3CL-protease)	Egyptian propolis	-	[60]
13-epi-torulosal, isoagatholal, and manool	n-butanol	Antiproliferative activity in HT-29 human colon adenocarcinoma cells	Greek Propolis	NMR	[61]
Gallic acid, caffeic acid, vanillin, p-coumaric acid, t-ferulic acid, benzoic acid, quercetin, t-cinnamic acid, naringenin, luteolin, genistein, kaempferol, apigenin, chrysin, pinocembrin, galangin and CAPE	Ethanolic extract	Improve human sperm motility	Czech propolis	HPLC	[62]
Ferrulic acid, p-coumaric acid, tectochrysin, galangin, pinocembrin pinocembrin-7-metylether, chrysin, apigenin, kaempferol	Methanol extract	Antioxidant and antimicrobial activity	Croatian propolis	HPLC	[63]
2-furanmethanol, cyclopentanedione, benzyl alcohol, 2-methoxyphenolacetat, benzoic acid, o-hydroxy-cinnamic acid, 2-hydroxy cinnamic acid, 2-methoxy-4-vinylphenol, 4-vinyl-2-methoxy-phenol, 4-hydroxybenzaldehyde, 4,5-dihydroxy-2-methyl-benzaldehyde, cinnamic acid, 4-hydroxy-acetophenone, 4-propyl-guajacol (=2-methoxy-4-propyl-phenol), cinnamic acid ethyl ester, 4-hydroxy-3-methoxyphenyl-2-propanone, 4-methoxyphenyl propanoic acid ethyl ester, dodecanoic acid ethyl ester, benzoic acid benzyl ester, myristic acid ethyl ester, salicylic acid benzyl ester, hexadecanoic acid, hexadecanoic acid ethyl ester, benzyl cinnamate, 9-octadecenoic acid ethal ester, stearic acid ethyl ester, 2′,6′-dihydroxy-4′-methoxy-chalcone, cinnamyl Cinnamate, 4-acetyl-benzoic acid phenylmethyl ester, caffeic acid, pinocembrin, and cinnamic acid	Ethanol and water	Antioxidant and antimicrobial activity	German Propolis	GLC/MS and HPLC	[64]
Benzyl alcohol, 2-phenyl ethanol, benzoic acid, glycerol, vaniline, cinnamic acid, 4-hydroxybenzoic acid, 6-hydroxy-β-caryophyllene, γ-eudesmol, β-eudesmol, benzyl benzoate, (Z) p-coumaric acid, α-fructofuranose, β-fructofuranose, α-mannofuranose, α-glucopyranose, (E) p-coumaric acid, β-glucopyranose, 3,4-dimethoxycinnamic acid, hexadecanoic acid, Isoferulic acid, ferulic acid, (E)-caffeic acid, 2-methyl-2-butenyl-(E)-p-coumarate, 3-methyl-2-butenyl (E)-p-coumarate, oleic acid, stearic acid, 2(3)-methylbutanyl-(E)-caffeate, 3-methyl-3-butenyl-(E)-caffeate, 2-methyl-2-butenyl-(E)-caffeate, 3-methyl-2-butenyl-(E)-caffeate, eicosanoic acid, pinocembrin, mono-TMS, 2′,6′,α-trihydroxy-4′-methoxychalcone, Benzyl p-coumarate-(E), pinocembrin chalcone, pinocembrin, pinobanksin, chrysin, mono-TMS, benzyl-(E)-ferulate, pinobanksin 3-acetate, benzyl (E)-caffeate, chrysin, di-TMS, 5,7-dihydroxy-3-methoxyflavanone, di-TMS, galangin, tri-TMS, pinobanksin 3-isobutanoate, 2-phenylethyl (E)-caffeate, CAPE, Isosakuranetin, 5,7-dihydroxy-4′-methoxyflavanone-TMS, tetracosanoic acid, pinobanksin 3-n-butanoate, Sakuranetin chalcone, sakuranetin, acacetin, 5,7,4′-trihydroxy-3′-methoxyflavanone, kaempferol, 6-hydroxy-β-caryophyllene p-coumarate, 14-hydroxy-β-caryophyllene, p-coumarate, 1,3-di-p-coumaroyl glycerol, tri-TMS, 2-acetyl-1,3-di-p-coumaroyl glycerol, TMS	Ethanol	antiproliferative effect glioblastoma	Polish propolis	GC-MS	[65]

Table 1 displays a variety of propolis types and their most influential constituents on the biological activity of propolis. Moreover, rather than bee species, the extraction method and solvent had a significant impact on the propolis compounds obtained. Ethanol solvent is commonly used in the extraction to isolate the major component in propolis [45,66-68]. However, the conventional extraction process by ethanol solvent has limitations such as the potential for harming the environment, poor extract quality, residual taste, and long extraction time. Thus, some researchers suggested supercritical fluid extraction with CO_2_ and ethanol as the co-solvent for propolis preparation [47,59]. This method was an appropriate step in generating propolis extract on a large scale. Furthermore, chromatographic analysis for propolis identification altered the results. By using the same supercritical fluid extraction method, the propolis compounds obtained from propolis extract (Trigona sp., Indonesia) and red Brazilian extract appear to be distinct, with the following flavonoid compounds are abundant in propolis, according to HPLC analysis, while volatile compounds dominate propolis, according to GC-MS/MS results. [47,59].

Each propolis' biological function is greatly influenced by its constituents. Yuan Min *et al*. [69] investigated the anti-inflammatory properties of Chinese and Brazilian propolis. HPLC study revealed that they contain identical chemicals, with the exception of artepillin C of Brazilian propolis. According to the study, both propolis have almost equivalent effectiveness in decreasing proinflammatory cytokines and antioxidant activity [69]. The cytotoxic effects of propolis from Turkey, Argentina, and China against nonaggressive and aggressive breast cancer cell lines have been reported to be comparable to their anti-breast cancer activity. This corresponded to the number of identical propolis components found in each sample [55]. In contrast, Propolis from stingless bees in Indonesia, results in a variety of biological activities and components. Propolis from *Tetragonula* aff. *Biroi* and *Tetragonula* sp. Sulawesi Indonesia, for example, contained distinct compounds and had varied effects (Table 1).

**Figure 1.**
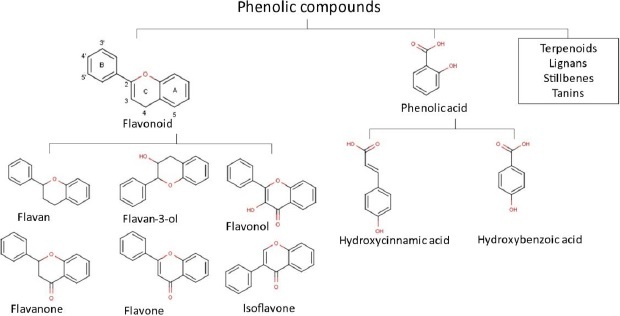
Chemical structures of flavonoid and phenolic groups of common propolis compound

Overall, almost all compounds found in propolis showed promising pharmacological effects for health and the diversity of their compounds results in a wide range of health benefits. In the majority, flavonoids, terpenoids and phenols are the most important compounds in propolis. Until the 2000s, over 300 flavonoid components, phenolics and terpenes were found in propolis. Moreover, from 2000-2014, a total of 241 new propolis compounds in a group of flavonoids, terpenoids and phenolics were identified [70]. Our previous studies also identified numerous flavonoid and phenolic acid compounds in propolis extract [71-73]. We have also explored 658 Asian propolis compounds that are majorly in the group of flavonoids, phenolic acid, and terpenoids [74]. The chemical structures of flavonoids and phenols group are presented in Figure 1.

### Physical and chemical properties of propolis

Previous studies show that propolis components consist of flavonoids, esters, aromatic acids, terpenes, alkaloids, alcohols, fatty acids, aliphatic acids, sugars, vitamins, minerals and other organic substances. Due to its high quantities of organic compounds, propolis has the characteristics of low bioavailability and low water solubility. It may be caused by organic compounds, which are often hydrophobic in nature [75,76]. There are several aspects that require enhancement of propolis in order to augment the efficacy of propolis treatment. Nevertheless, the propolis products for herbal supplements are massively produced by various industries, for example, EPP-AF®, Actichelated® Propolis, Propolis Lenigola Spray Forte, Propoelix®, Bio Propolis defense APF® 10 spray, organic propolis extract-Hoyer, Propolia, Bee & You water-soluble propolis and many other [77-80]. These products formulated propolis in medical preparations with improved bioavailability and medical effectiveness. Actichelated® Propolis was the first aqueous commercial product of propolis. In Europe, almost all propolis products are water-based and alcohol-free, such as Bio Propolis defense APF® 10 spray, Propolia, and Bee & You water-soluble propolis. EPP-AF® was the only chemically and biologically characterized propolis-based product on the market. Moreover, Propoelix ® is produced by the superextraction process and removing wax and resins, which results in propolis in a water-soluble form [77-80]. As valuable insight for the use of propolis in medications, besides its low solubility, bioactive compositions of propolis extract were inconsistent through the different seasons, vegetations and environmental conditions. Chemical and biological characterization of each type of propolis must be obtained. Furthermore, the high wax content of propolis must be removed during the extraction process to avoid consumption. The process of eliminating wax from propolis not only ensures its purity but also results in the reduction of unwanted fat-soluble compounds, such as drug residues [81].

Despite significant research into propolis’ biological effects and traditional knowledge of propolis consumption among the community, the precise targets and pharmacokinetics study of propolis still remain unknown. Those factors were connected to the research outcomes that demonstrated inconsistent medical results regarding the efficacy of propolis for diabetes treatment [82]. The utilization of nanoformulations can lead to an improvement in the bioavailability, solubility, and pharmacological activity of propolis, resulting in better medicinal properties such as increased precision in targeting and a more specific mechanism of action. Nanoformulation products from propolis extract are starting to be developed and marketed. The inherent properties of propolis can be exploited in a controlled and targeted manner by incorporating it into nanocarriers, such as nanoparticles, liposomes, micelles, or nanofibers. Its potential use can also be used as a natural food additive.

## Current nanoformulations of propolis

Propolis is a bee product with several pharmacological activities, such as antioxidant, antimicrobial, antifungal, antiviral, anti-parasite, anti-inflammatory, and wound healing. Along with its hydrophobic nature, propolis is not very well absorbed by the body. Formulation of the propolis by using nanotechnology is expected to increase its bioavailability, solubility, adsorption, and target release.

Nanoformulation of herbal extracts can be accomplished using various techniques [83,84]. Generally, there are two approaches for the preparation of nanopropolis, *i.e.* the top-down and bottom-up approaches. In the first approach, nanopropolis is obtained by gradually decreasing the dimension of propolis into nano-sized particles. For instance, Sahlan *et al.* [85] reported nanopropolis preparation by encapsulating propolis extract in casein micelle and reducing their size with a high-pressure ball mill homogenizer. As a result, nanopropolis with a particle size of 80 nm and an encapsulation efficiency of 80 % was obtained [85]. Ay *et al.* [86] prepared nanopropolis using sonication method. A propolis extract was diluted 1:10 with distilled water, then sonicated for 1 h and filtered through a 220 nm filter. As a result, nanopropolis with an average particle size of 59.28 nm (PDI: 0.507) was obtained [86]. The same method was also used by Kazemi *et al.* [87]. The lyophilized propolis extract was dissolved in 70 % alcohol and sonicated for 20 min. The findings revealed that the morphology of the propolis nanoparticles was spherical, with a diameter of less than 100 nm [87].

In bottom-up approach, nanopropolis is commonly prepared by emulsification, precipitation, or encapsulation methods. One example of a bottom-up approach to nanopropolis preparation was reported by Elsharkawy and Elsherif [88], who used a spontaneous emulsification method with the low-energy formation of propolis nanoemulsion. A fine powder of pure propolis was added to double-deionized distilled water at a ratio of 2:1 w/v. This oil phase was then added to the water phase containing hydrophilic surfactant Tween 80 at a concentration of 3 %. The mixture was maintained on a magnetic stirrer at 3000 rpm for 7 h at 40 °C. Afterward, the solution was sonicated for 10 minutes and filtered with a 200 nm nano-filter [88]. Nascimento *et al.* [89] prepared nanopropolis by nanoprecipitation. The suspension of propolis nanoparticles was obtained by adding the organic phase (composed of propolis extract and polycaprolactone) into the aqueous phase (composed of pluronic) and then vortexed for 1 min. As a result, a homogeneous suspension with an opaque appearance was obtained, in which the particle size of the suspension varied between 208.5 and 280.2 nm [89]. An example of nanopropolis preparation by encapsulation method was reported by Soleimanifard *et al.* [90], in which sodium caseinate-maltodextrin nanocomplex was used as the encapsulant . Propolis extract solution was added dropwise to sodium caseinate solution at various ratios, followed by stirring and homogenizing in an ultrasonic bath. Maltodextrin was used as an assistant drier. The suspension was then centrifuged and freeze-dried to obtain propolis nanocomplex.

In recent years, several studies have reported nano-formulation of propolis, particularly for biomedical applications, through the incorporation of propolis into electrospun nanofibers. Electrospinning is a technique for producing nanofibers (with diameters ranging from a few to a hundred nanometers), achieved by applying high voltage to induce polymer melting and form fibers. For example, Kim et al. [91]. Reported preparation of propolis-loaded polyurethane nanofiber using electrospinning process. 10 wt% polyurethane solution containing various concentrations of propolis was electrospun at room temperature, with a 17 kV applied voltage, a tip-to-collector distance of 14 cm, and a solution feed rate of 0.5 ml/h. The resulting nanofibers were characterized for their physicochemical properties, in vitro cell compatibility, and in vitro antibacterial activity. The results indicated that blending propolis with polyurethane led to improved physiochemical and biological characteristics. Similar studies on propolis nanofibers preparation were also reported, but using different polymers such as polyvinyl alcohol [92,93], polycaprolactone [94], and poly(lactide-co-glycolide) [95].

Apart from those described above, there are many more methods for propolis nanoformulation, a resume of which is depicted in Table 2. Various types of propolis nanomaterial have been reported, including propolis encapsulated in polymeric material, nanoemulsion, liposome, nanosuspension, niosome, nano-structured lipid carrier, nanocomposite, NIM (nano in microparticle), solid lipid nanoparticle, nanofiber and others (Table 2). Among them, chitosan and polyvinyl alcohol are often used as encapsulants or nanocarriers as polymeric structures that entrap, coat or encapsulate propolis particles to obtain propolis nanoforms. This is due to the properties of chitosan, which has a wide range of biological activities, such as non-toxic, biodegradable, antibacterial activity, wound healing and biocompatibility, which can be combined with polymers, metals, and ceramic materials. It is also employed for material-body contact because the free amino groups in cationic charge promote interaction with the anionic cell surface [96,97]. PVA is a water-soluble polymer that is also biocompatible, biodegradable, and capable of enhancing oxygen permeability.

**Table 2.** Various methods used for obtaining nanopropolis with the size’s result.

Application	Type of bionanomaterial	Synthesis methods	Composition	Size, nm	Ref.
Anti-diabetes	Nano-spheres powder of extract ethanol propolis encapsulated chitosan-polyacrilic nanoparticles	Chitosan-poly acrylic acid (CS-PAA) polymer was synthesized by converting chitin to chitosan through a deacetylation process, followed by mixing the chitosan solution with polyacrylic acid and stirring overnight. Then, ethanolic extract of propolis (EEP) was conjugated with the CS-PAA polymer using a a home-made rod mill, then sonicated, and centrifuged	Chitosan, ethanol extract of propolis, 0.66 mM potassium persulphate (KPS), acrylic acid, 0.1 N hydrochloric acid (HCl)	24-32	[99]
	Nanopropolis lyophilized powder	Extract ethanol propolis was lyophilized and dried	Ethanol extract of propolis, ethanol	<100	[87]
	Nanopropolis emulsion	Spontaneous emulsification method. Dissolving propolis extract into an organic phase containing soy lecithin ad mix with aqueous phase containing poloxamer	0.4 % of poloxamer, 2.5 mg/mL of soy lecithin, acetone and ethanol with ratiof 60:40	122.1	[100]
Hepatoprotective	Nanopropolis polymeric micelles solution	Micellar formulation method. Adding propolis extract to triblock copolymer solution and evaporated to remove ethanol	0.06734 g ml^-1^ PEO_26_PPO_40_PEO_26_ triblock copolymer, 0.055 g ml^-1^ of propolis extract, ethanol:water (70:30)	21	[101]
	Propolis liposome	All materials were mixed with ethanol and water while heated in 80 °C. Then, the solution was homogenized in ultra-turrax homogenizer at 11000 rpm. Then, the liposome dispersion was obtained.	160 mg of phospholipids (PL-90H), 80 mg of cholesterol, 100 mg stearic acid, 30 mg of propolis extract, and ethanol	216 to 437	[102]
Anti-cancer	Chitosan-coated nano-propolis	Green sonication method by adding chitosan into acetic acid solution then sonicated. Tween 80 was added and mixed with propolis ethanol solution. Subsequently, the solution was sonicated and stored in cold.	3.5 g chitosan, 2 vol.% acetic acid, 1 g of tween 80, propolis (85 %), 120 mL ethanol	<200	[96]
	Propolis nanosuspension	Nanoprecipitation method by dissolving a half of total propolis to DMSO 5 % and the rest in ethanol, then it was sonicated. Propolis solutions added to distilled water containing lecithin, while in stirring. Nanosuspension obtained after ethanol evaporated	5 % dimethylsulfoxide (DMSO), 2 ml lecithin, ethanol, distilled water, and propolis extract	185.27 ± 26.44	[103]
	Niosome vesicle of propolis	Ether injection method in synthesizing niosomes. Diethyl ether solution was prepared and dissolving cholesterol and tween 80, then propolis extract in PBS solution added to the mixture. Niosome was obtained by centrifugation and lyophilized in extreme cold temperature	Cholesterol, tween 80, diethylether, PBS pH 7.4 and propolis extract	151 ± 2.84	[104]
	Nanoparticle propolis	Propolis extract dissolved in ethanol and shaking in water bath. Then it was filtered and diluted in distilled water. Propolis water solution sonicated and filtered using a 220nm PTFE filter	Ethanol, water and propolis powder extract	43.82 to 91.28	[86]
	Propolis-loaded nanostructured lipid carrier	High-shear homogenization. A lipid phase mixed with propolis ethanol solution and heated in water bath above melting point of lipid. Aqueous phase was prepared containing emulsifier. Lipid and aqueous phase mixed with Ultra Turrax homogenizer at the same temperature	Liquid lipid, Poloxamer 188, Poloxamer 407, ethanol, propolis extract	255.8 to 342.5	[105]
	Zinc Oxide-Propolis Nanocomposite	Propolis nanoparticle prepared by ball mill method and Zinc oxide nanoparticle obtained by coprecipitated using zinc nitrate and propolis aqueous extract. Then, ZnO-Propolis Nanocomposite synthesize by green synthesis approach by mixing both propolis and ZnO nanoparticle and centrifuged in 4 times	zinc nitrate, milli-Q water, propolis	9.70	[106]
	Propolis-loaded nano-in-microparticles	Propolis extract solid dispersion loaded to polymeric materials such as poly(lactic-co-glycolic acid) (PLGA) and poly(ethylene glycol)-block-poly (propylene glycol)-block-poly(ethylene glycol). PE loaded polymer were dispersed in chitosan solution formed NIM (nano-in-microparticles)	polyvinyl alcohol, poly(ethylene glycol)-block-poly (propylene glycol)-block-poly(ethylene glycol), PLGA, polyethylene glycol, gelucire, Sodium dodecyl sulfate	104 to 1921	[107]
	Propolis solid lipid nanoparticles	Hot homogenization method and ultrasonication. Propolis nano-emulsion placed in cold water to obtain solid lipid nanoparticles	Tween20, Tween80, solid lipid, ethanolic extract of propolis.	57.55 ± 15	[108]
	Propolis nanocapsule	Ultrasound and high-speed homogenizer method. First, propolis extract in aqueous solution prepared and adding water gradually in stirring. High speed homogenizer used to obtain nanoemulsion. sodium alginate added to nanoemulsion then ultrasonicated to obtain encapsulation emulsion	supercritical fluid carbon dioxide extract of propolis, deionized water, sodium alginate	18.87to 39.12	[109]
Cardiovascular disease	Liposomal propolis	Liposome obtained by modification on dried thin lipid film technique. Then, propolis extract in ethanol mixed with liposome and vortexed. Propolis liposome obtained by evaporating ethanol solvent.	0.25gram L-α phosphatidylcholine, 0.25gram cholesterol. 15 ml chloroform, phosphate buffer, ethanol, propolis extract.	93.5 ± 0.87	[110]
	Propolis loaded triblock copolymer nano-vesicles	Thin film hydration method	Polylactide-block-poly (ethylene glycol)-block-polylactide (PLA-PEG-PLA), ethanol, propolis extract	23-53	[111]
Anti-virus	Propolis-loaded chitosan/PLGA	oil-in-water (o/w) single emulsion solvent evaporation method	PLGA, PVA, chitosan, Dichloromethane, ethanolic extract of propolis	450	[112]
	Propolis-loaded PLGA nanoparticle	oil-in-water (o/w) single emulsion solvent evaporation method	PLGA, PVA, Dichloromethane, ethanolic extract of propolis	229.5 to 424.7	[113]
	Propolis-chitosan nanoparticle	Chitosan nanoparticle prepared by ionic gelation method using tripolyphosphate solution and acetic acid. Then, chitosan was added to ethanolic propolis extract with tween 20 using peristaltic pump. while stirring	0.1% (w/v) chitosan, tripolyphosphate, 1% glacial acetic acid, ethanolic propolis extract, Tween 20	50 to 200	[97]
	Propolis nanofiber	Electrospinning method and fabrication of nanowebs	6 wt% PVA, propolis glycerin extract, propolis extracts containing ethyl alcohol	>5000	[92]
Antibiotics, antibacteria	Chitosan propolis nanoparticle	Ionotropic gelation method with Tripolyphosphate (TPP). 0.2% Chitosan solution was prepared in 1% acetic acid at ph 5.0. Then, ethanol extract of propolis added while stirring. TPP solution prepared in distilled water and adjusted to ph5.5. chitosan-propolis solution added into TPP under stirring condition	0.2% w/v of chitosan, 0.15% w/v sodium TPP, 1% v/v acetic acid, ethanol extract of propolis	107.74 ± 0.53	[114]
	Nanopropolis	Ball mill media method using ball or rods	Propolis extract and methanol	122 to 409	[115]
	Propolis nanoemulsion	Chitosan solution prepared in 1 % lactic acid then mixed with 0.4 % TPP solution. Suspension obtained by stirring in overnight. 35 % of glycerol-sorbitol solution (2:1) added to suspension. Propolis extract was added to form nanoemulsion	Chitosan, 1 % lactic acid, 0.4 % TPP solution, 35 % of glycerol-sorbitol solution (2:1), propolis extract	71.16 to 112.34	[116]
	Propolis- nanostructured lipid carrier	Emulsion-evaporation-solidification method. Propolis extract dissolved in lipid phase with lecithin in ethanol and heated. Tween 80 in water as aqueous phase mixed with lipid phase in dropwise and then sonicated	Glycerol monostearate, capric acid, lecithin, ethanol, tween-80, propolis extract	41.57 to 44.28	[117]
	Propolis micellar nanocomposite	A combination of polymeric nanocomposite mixed with propolis ethyl acetate extract. Then, acetone used for solubilize the micellar nanocomposites loaded propolis	poly-ε-caprolactone (PCL), Pluronic F108 copolymer, acetone propolis ethyl acetate extract	>70	[118]
	PVA-loaded nanopropolis	PVA solution was prepared and mix with propolis extract while shaking at 80 °C for 1 h. The solution was continued homogenized in 24 hour using magnetic stirrer. The sample weas filtered and put into electrospray to obtain PVA-nanopropolis	6 % of PVA, Propolis extract	106.37 to 258.51	[119]
Anti-inflammation	Propolis nanoparticles	Emulsion diffusion method. Propolis in 90 % ethyl alcohol was added drop wise into polyvinyl alcohol (PVA) in aqueous solution under stirring. Then propolis nanoparticle obtained by centrifugation	1 % of polyvinyl alcohol (PVA), ethyl alcohol, propolis, distilled water	5 to 10	[98]
	Propolis supercritical extract encapsulated in gamma- cyclodextrin	Propolis extract mixed with cyclodextrin in aqueous solution and homogenized. Then, suspension was dispersed in water and spray-dried	Green propolis supercritical extract, gamma cyclodextrin, water	<6000	[120]
Oral and dental disease	Colloidal nanopropolis	Propolis extract was filtered and added to distilled water. The suspension was sonicated to obtain colloidal nanopropolis. pH of nanopropolis adjusted to 7 with natrium hydroxide. The result was then powdered using freeze-drying process	Propolis, ethanol, natrium hydroxide	70 to 120	[121]
	Niosomal propolis extract	Reversed phase evaporation method	Span60, cholesterol, chloroform, ethanol, water, propolis extract,	237 to 333	[122]
Wound healing	Nanofiber propolis-silk fibroin gelatin	Silk fibroin was prepared first from silk cocoons using degumming and lyophilization method. Gelatin mixed with silk fibroin and propolis extract was added. Then, solution was processed into nanofiber using electrospinning method.	silk cocoons, lithium bromide, water, formic acid, gelatin,	100 to 600	[123]
	Nanofiber propolis gel patches	Electrospinning method. 3 % of gelatin added to PVA solution and mixed with tween 80 and propolis. Then the solution introduced to electrospinning	PVA, Tween 80 propolis, gelatin	293 to 401	[93]
	Nanofiber propolis	Electrospinning method. Propolis was mixed with polymeric solution and 1,1,1,3,3,3-hexafluoro-2-propanol was used as solvent. Then, the mixture introduced to electrospinning	poly(lactide-co-glycolide), propolis,	n/a	[95]
	Propolis loaded cellulose nanofiber /polyvinyl alcohol hydrogel	Freeze-thawing and freeze-drying method	PVA. Cellulose nanofiber, propolis extract, distilled water,	<20000	[124]

The synthetic polymer PVA is nontoxic and non-antigenic, which makes it a popular choice for nanocarriers [93,98]. Besides them, PLGA and triblock copolymers are also used as nanocarrier materials. Lipid materials such as cholesterol, phosphatidylcholine, lecithin, and glycerol monostearate are commonly used as nanocarrier-based liposomal or nanoemulsion, along with using surfactant materials, such as tween 20, tween 80, span 80, poloxamer, and others. Various methods have been reported as principal methods to obtain nanopropolis, such as nanoencapsulation, nanoprecipitation, high-shear homogenization, thin film hydration, single emulsion solvent evaporation, electrospinning method, Ionotropic gelation, ball mill method, emulsion-evaporation-solidification method, emulsion diffusion method, reversed-phase evaporation method, freeze-thawing and freeze-drying method (Table 2). These propolis nanoparticles have numerous superior activities to combat various human diseases. This will be described in the next session of this article.

## Pharmacological actions of nanopropolis in various diseases and conditions

### Diabetes mellitus

Diabetes mellitus is a chronic condition characterized by prolonged hyperglycemia. Previous studies show that this condition can cause increased oxidative stress due to an imbalance between antioxidants and increased levels of reactive oxygen species (ROS) [125]. As a result, this imbalance causes insulin resistance by inducing mitochondrial fission, with this resistance directly linked to skeletal muscle [126,127]. The potential benefits of propolis have been proven in previous research, where the ethanol extract of propolis increases endogenous antioxidants to combat the pro-oxidant actions of ROS through the Erk/Nrf2/GCLM, HO-1, and TrxR1 signaling pathways [128,129]. Moreover, there is evidence showcasing the effectiveness of propolis in diminishing oxidative harm in diverse tissues of diabetic rats induced by alloxan or streptozotocin. Propolis plays a role in regulating oxidative stress, the buildup of advanced glycation end products (AGEs), and inflammation in adipose tissue—factors that collectively contribute to insulin resistance or hinder insulin secretion. Additionally, propolis has demonstrated efficacy in mitigating diabetes-related complications, including nephropathy, retinopathy, diabetic ulcers, and non-alcoholic fatty liver disease [130,131]. This beneficial effect is mediated by the antioxidant activity of propolis, which aids in the detoxification process in the body and has a protective effect on humans. Furthermore, nanotechnology has been used to maximize the benefits of propolis activity and has shown promising potential in several studies.

Based on the results of Sarah's research, using Egyptian propolis ethanol extract conjugated with polyacrylate chitosan nanoparticles showed a potential therapeutic effect on type 2 diabetes mellitus by significantly decreasing blood glucose levels in diabetic test mice [99]. Another report, the Iranian nanopropolis ethanol extract, was reported to have anti-glycation effects by inhibiting the formation of AGEs and preventing the degradation of heme Hb during glycation. Without significant changes, the secondary structure of Hb remains unchanged when exposed to glucose, fructose, and nanopropolis [87]. Nanopropolis show superior reactivity compared to bulk propolis due to the substantial increase in the surface-to-volume ratio of the nanoparticles as their size decreases. nanopropolis, which has strong antioxidant characteristics, has the potential to function as a more efficacious anti-glycation agent in terms of volume when compared with propolis. Clinical trial data suggest that although aspirin exhibits moderate anti-glycation effects when compared with propolis, 21.5% of diabetic patients may experience resistance to its effects, potentially resulting in cardiovascular complications. Another report also stated that the ethanol extract of nanopropolis from Southern Brazil provided a therapeutic effect for topical wound healing caused by diabetes mellitus by showing a better percentage of wound closure (54.5%), whose mechanism was supported by nanopropolis from Southern Brazil in inducing acceleration of the proliferation phase, collagen deposition, angiogenesis, thereby forming new skin tissue regeneration [100]. Finally, nanopropolis formulated with powder effectively treated diabetes by reducing blood sugar levels and regenerating damaged β cells in streptozotocin-induced diabetic mice [132]. While research on anti-diabetic activity is still limited, these findings suggest that nanopropolis may have the potential to be a natural drug candidate for diabetes treatment.

### Hepatic disease

Propolis is also known for its hepatoprotective activity, supported by several studies demonstrating its ability to shield the liver from oxidative stress and injury caused by CCL_4_ [133]. Additionally, the mechanism underlying its hepatoprotective effect plays a role in reducing liver cell DNA damage in vitro, displaying antineoplastic activity in human hepatocellular carcinoma cells. It has no adverse effects on normal cells and can reduce liver enzymes and the severity of cirrhosis caused by thioacetamide [129]. Tzankova's research on Bulgarian propolis micellar nanoformulation also revealed hepatoprotective activity in nanopropolis. The study demonstrated liver protective effects, including the restoration of glutathione levels, normalization of serum transaminase activity, significant protection against the liver antioxidant enzymes SOD and CAT, and histopathological analysis showing good hepatoprotection and improved liver injury acutely induced by CCL_4_ in test mice [101]. Furthermore, the hepatoprotective activity of nanopropolis ethanol extract from Indonesia was identified. The flavonoid content in nanopropolis demonstrated a reparative effect on liver cells. The results indicated that the nanopropolis group with a concentration of 56 ppm exhibited a relatively good hepatocyte condition, with visible and radially arranged hepatocytes, although fat granules were still present [134]. Lastly, the European propolis ethanol extract of nanopropolis was reported to have more effective hepatoprotective activity in suppressing rat-induced levels of AST, ALT, and ALP biomarkers, promoting tissue healing [102]. While there are still limited reports testing nanopropolis against liver disease, considering these findings, and nanopropolis holds promise as a hepatoprotective agent.

### Cancer disease

The potential for utilizing the natural medicinal ingredient propolis as an effective cancer treatment shows great promise [135,136]. It is important to note that contemporary cancer therapies, including chemotherapy and radiation, can cause significant side effects [137]. Therefore, it is critical to accelerate the development of new cancer treatments demonstrating improved efficacy and safety. Nanopropolis has been widely reported for its use as a cancer therapy. A previous study reported that nanopropolis has efficacy in ameliorating severe hepatotoxicity and nephrotoxicity caused by drugs at a dose of 30 mg/kg [96]. In addition, other research suggests that nanopropolis exerts a notable inhibitory influence on tumor development in female mice. This effect is attributed to the heightened activity of superoxide dismutase (SOD) and increased glutathione content observed in the liver and EAC cells. These outcomes are thought to result from the prevention of oxidative damage, immune stimulation, and the induction of apoptosis [103]. In Table 3, we have comprehensively summarized the activity of various types of cancer from different Propolis and solvents.

**Table 3.** Anticancer activity of various types of nanopropolis

Types of propolis	Types of cancer	Solvent	Findings	Ref.
Indonesia propolis	Breast	Ethanol	Administering nanopropolis at a dose of 32 μg ml^-1^ and Propolis at a dose of 233 μg ml^-1^ produced comparable effects in reducing tumor size, promoting the healing of tumor-related wounds, and eliminating breast cancer cells	[138]
Turkey propolis	Lung	Ethanol	Propolis-loaded niosomes exhibited substantial cytotoxic effects, demonstrating a significant reduction in viability and a fivefold increase in cell spreading compared to untreated cells	[104]
Egyptian propolis	Breast	Ethanol	Nanopropolis showed significantly higher cytotoxic and apoptotic effects (*p* < 0.0001) on cancer cells compared to healthy cells and Propolis	[86]
			NLC-propolis demonstrates anti-breast cancer activity by increasing antioxidant levels, suppressing angiogenesis, inflammatory and mTOR pathways, and inducing the apoptotic pathway. This leads to an increase in the expression of miRNA-223, contributing to the suppression of breast cancer	[105]
		Water	The composite of ZnO and propolis (ZnO-P NCs) exhibited strong cytotoxic effects on breast cancer cells, displaying an IC_50_ value of 18 μg/mL, indicating its potential for the treatment of breast cancer	[106]
	Cervical		ZnO-P NCs shows cytotoxicity against cervical cancer with an IC_50_ value of 23 μg/mL	
	Liver	Ethanol	In vitro cytotoxicity studies revealed that nano-in-microparticles containing propolis induced more significant cytotoxic effects on HepG2 cells, exhibiting a threefold higher therapeutic effect than free propolis and NIMs. Propolis induced apoptosis in HepG2 cells, significantly reducing their numbers in the G0/G1, S, and G2/M phases of the proliferation cycle	[107]
Iranian propolis	Colorectal	Dimethyl sulfoxide	Combination between propolis and layered double hydroxide nanoparticles notably boosted anticancer capabilities by elevating the expression of the pro-apoptotic gene Bax, reducing the expression of the anti-apoptotic gene Bcl-2, and initiating apoptosis	[139]
	Lung	Ethanol	Solid lipid nanoparticles (SLNs) loaded with propolis demonstrated notable anti-cancer characteristics against the A549 cell line, exhibiting an IC_50_ of 0.01 mg/ml following a 72-hour treatment period	[108]

### Cardiovascular disease

To date, reports on the use of nanopropolis in cardiovascular disease therapy are very limited. However, we have identified two important reports related to this therapy. The administration of the Iranian nanopropolis formula has demonstrated efficacy in reversing cardiac tissue damage caused by DOX by reducing serum concentrations of CK-MB, Troponin I, and Lactate dehydrogenase [110]. Lastly, Chinese nanopropolis exhibits cardioprotective effects by reducing inflammation and stimulating the SIRT1-AMPK axis [111]. Although discussion of this therapy is still very limited, these findings indicate that nanopropolis holds great promise in treating cardiovascular diseases.

### Viral infections

The antiviral properties of nanopropolis have also been reported based on previous research findings. The ethanol extract of Thai propolis (EEP) encapsulated in nanoparticles showed reduced cytotoxicity in vero cell assays and directly impacted viral particle inactivation. The role of nanopropolis is to prevent viral particles from entering or leaving host cells by acting as a barrier. In addition, the expression levels of HSV replication-related genes, especially ICP4, ICP27, and gB, have decreased significantly [112,113]. Uniquely, the use of nano propolis-chitosan in treating NDV infections, although it produces low cytotoxicity, shows promising antiviral activity against NDV infections [97]. Finally, propolis nanofiber from three propolis sources (Brazil, Latvia, and Lithuania) showed antiviral effects against SARS-CoV-2 (more than 2 logs) and was not cytotoxic [92]. Unfortunately, literature regarding the use of nanopropolis as an antiviral is still limited.

### Bacterial infections

Numerous researchers have conducted investigations into the synergistic antibacterial capabilities of propolis. In most *in vitro* and *in vivo* trials, bacteria's resistance to conventional antibacterial drugs was notably diminished [140]. Propolis and its derivative compounds are commonly acknowledged to possess antibacterial properties. Notably, the compound Quercetin has been found to exhibit antibacterial effects against various strains, including MRSA, *Staphylococcus epidermidis*, *Bacillus subtilis*, *S. aureus*, and oral bacteria such as *Porphyromonas gingivalis* [141-143]. Furthermore, several constituents of propolis, including aldehydes, aliphatic acid esters, carboxylic acids, and cinnamic acids, and their esters, ketones, terpenes, alcohols, ethers, hydrocarbons, and phenolics, have been found to possess antibacterial effects [144]. Following this, extensive evaluations have been conducted on the antibacterial effectiveness of nanopropolis and its specific impacts. The present investigation involved the analysis of data about the antibacterial efficacy of nanopropolis against a diverse range of bacterial strains, as presented in Table 4.

**Table 4.** The antibacterial activities of various types of nanopropolis

Types of propolis	Target bacterial strains	Effects	Ref.
Malaysian propolis	*S. epidermidis*	Chitosan-propolis nanoparticles effectively disrupted the formation of *S. epidermidis* biofilm and reduced viability by approximately 25 %, demonstrating superior efficacy in diminishing the viability of both planktonic bacteria and biofilms. At a concentration of 100 μg ml^-1^, CPNP decreased the viability of biofilm bacteria by around 70 %, and based on observations of the bacterial biofilm's structure, CPNP notably reduced the number of bacteria in the biofilm by approximately 90 %. This impact is influenced by intercellular adhesion, involving genes such as IcaABCD, embp, and others, which CPNP significantly suppresses. Lastly, CPNP synergizes with the antibiotics rifampicin, ciprofloxacin, vancomycin and doxycycline.	[145]
	*E. faecalis*	Chitosan-propolis nanoparticle exhibited a significantly higher efficacy in decreasing *E. faecalis*. Electron microscopy images also showed greater effectiveness of CNP in reducing *E. faecalis*	[114]
Thailand propolis	*C. albicans*	The compound efficiently inhibits the virulence factors of *C. albicans*, such as adhesion, hyphal germination, biofilm formation, and invasion, at concentrations of 1 and 2 μg ml^-1^.	[113]
Indonesia Propolis	*E. coli*	The assessment of antibacterial activity reveals that nanopropolis at concentrations ranging from 10 to 0.02 % has efficacy against *E. coli*, effectively inhibiting its growth even at deficient concentrations.	[146]
Iranian propolis	*C. albicans* and *S. aureus*	Propolis and nanopropolis significantly differed in their inhabitation zones against *S. aureus* (*p*<0.01) and *C. albicans* (*p*<0.05).	[115]
Korean propolis	*Staphylococcus aureus*, *Fusarium culmorum* and *Aspergillus parasiticus*	The antibacterial activity of propolis ethanol extract with chitosan nanoemulsion was seen at 50, 100, 150, and 200 ng concentrations. This activity was particularly noteworthy against pathogenic bacteria, including methicillin-resistant *Staphylococcus aureus* and *Fusarium culmorum*. The propolis ethanol extract chitosan nanoemulsion exhibited varying activity levels across four different concentrations, with the concentration of 150 ng demonstrating the highest degree of significance. Furthermore, when present at doses of 150 and 200 ng, it can effectively suppress the synthesis of zearalenone in Fusarium media, resulting in total inhibition of fungal growth. Furthermore, it has been observed to exert a suppressive effect on aflatoxin synthesis in aspergillus media, resulting in a notable reduction of mold growth by up to 47.18 %.	[116]
Egyptian propolis	*Bacillus subtilis, S. aureus, Salmonella spp.* and *Candida albicans*	Propolis-NLCs exhibited a 2-fold stronger inhibitory effect against bacterial microbes, including *Bacillus subtilis*, *S. aureus*, *Salmonella* spp. and *C. albicans* (*p* < 0.0001).	[117]
Brazilian propolis	*Streptococcus mutans, Lactobacillus acidophilus)* and *C. albicans*	EARP showed better antimicrobial efficacy, micellar nanocomposites containing ethyl acetate extract from Brazilian red propolis showed encouraging results in combat against the main causes of dental caries, namely *Streptococcus mutans* and *Lactobacillus acidophilus*, as well as *Candida albicans*	[118]

### Inflammation and immune system

Propolis, a natural resin produced by bees, is known for its broad range of biological properties, including immunomodulatory and anti-inflammatory effects. These effects have been established through in vitro and animal models investigations. A self-nano emulsifying drug delivery system (SNEDDS) incorporating propolis extract (PE) demonstrated a considerably more significant immunostimulant effect than common PE, according to a recent study [147]. Nitrite oxide production, phagocytic activity, and cell proliferation increased in RAW 264.7 cells in response to SNEDDS. Furthermore, it stimulated an upregulation of macrophage phagocytic activity and increased leukocyte, neutrophil, and lymphocyte populations [76]. Izzularab *et al.* [98] further investigated the application of propolis nanoparticles made from emulsion to treat nephropathy and liver fibrosis caused by carbon tetrachloride (CCl_4_) in an albino rat model. This medication showed anti-inflammatory properties, decreased the expression of nephrin in kidney tissue, and transformed growth factor β (TGF-β) in liver tissue. By upregulating Bcl-2 and downregulating Caspase-9 expression in liver and renal tissue, the propolis nanoparticles also demonstrated an anti-apoptotic impact [98]. In a third study, Rimbach *et al.* [120] investigated the anti-inflammatory activity of Brazilian green propolis supercritical extract (GPSE) encapsulated in β-cyclodextrin (CD) in the mouse liver in vivo in a third investigation. According to the findings, GPSE-CD dramatically reduced the mRNA levels of tumor necrosis factor and other pro-inflammatory indicators [120].

### Oral and dental diseases

Nano-propolis shows potential as an innovative therapeutic solution for various oral and dental issues. Formulations of nanopropolis offer several advantages compared to conventional propolis preparations, including heightened bioavailability, increased antimicrobial effectiveness, and enhanced tissue penetration. In a recent investigation by Zaleh *et al.* [121], the synergistic effects of propolis nanoparticles (NPro) and nano-curcumin-based photodynamic therapy (NCur-PDT) in remineralizing white spot lesions (WSL) were explored ex vivo. The study results indicated that the combination of NPro and NCur-PDT surpassed individual treatments, accomplishing enamel remineralization in a shorter duration. Different studies conducted by Arafa *et al.* [122] concentrated on creating oromucoadhesive films designed for the buccal administration of propolis extract (PPE) enclosed in niosomes for treating oral recurrent aphthous ulcers (RAU). This inventive method successfully diminished the size of ulcers, facilitated thorough healing, and alleviated pain . Additionally, Nassar *et al.* [148] conducted a study to investigate the impact of propolis and nano-propolis on the clinical outcomes of necrotic mature anterior teeth with apical periodontitis following regenerative endodontic treatment. The results of this study revealed that both propolis and nano-propolis were successful in promoting healing and the survival of the treated teeth.

### Wound healing

Lastly, because propolis contains beneficial bioactive compounds, it positively impacts various biological activities, including wound healing. Several studies have confirmed that using propolis in wound healing reduces healing time and accelerates the processes of tissue contraction and repair. This is supported by its antimicrobial and anti-inflammatory properties, mediated through various mechanisms, including the ability to halt reactive oxygen species (ROS), chelate metal ions and disrupt the series of reactions causing lipid peroxidation when combined with other antioxidants [147,148]. Additionally, propolis increases collagen production and accelerates the healing of diabetic wounds [149]. Nanopropolis is currently being explored for its potential in wound healing.

Propolis, when conjugated with chitosan biofilm nanoparticles, reduces the maturation time of granulation tissue and enhances full-thickness wound healing in mice with excision wounds. This is attributed to the reduction in neutrophil infiltration and normalization of macrophage influx to the injured area, leading to the disappearance of nodular shapes and quicker wound healing [150]. Another noteworthy finding is that Iranian nanopropolis, when conjugated with ZnO/Ag/Ext, accelerates wound healing and increases the wound closure ratio [151]. Furthermore, the ethanol extract of propolis nanoparticles in poly(-lactic-co-glycolic acid) was reported to significantly accelerate and improve wound healing by stimulating angiogenesis, fibroblast proliferation, and granulation tissue formation in the early stages of the healing phase. This accelerates the repair of diabetic wounds associated with earlier wound contraction and stability of the damaged area through the rearrangement of granulation tissue and collagen fibers [152].

Next, the electrospun propolis nanofiber (NFs) formulation significantly enhanced wound healing. In vitro test results indicated that propolis nanofiber has good cytocompatibility and blood compatibility, supporting cell adhesion and growth over an extended period. It also significantly encourages L929 cell migration. In vivo tests confirmed that propolis nanofiber accelerates wound healing, and histological experiments further verified its pro-healing properties [123]. Propolis-based nanofiber patches were also reported to improve corneal microbial keratitis by providing antimicrobial activity against *S. aureus* and *P. aeruginosa*, common microorganisms causing corneal keratitis. Propolis was also reported to be biocompatible with mesenchymal stem cells (MSCs) [93].

In other report, in the treatment of burns, propolis nanofibers had a beneficial effect on the regeneration of burn wounds at high levels of Fe^3+^ in lower transferrin signals after 10 and 21 days of therapy, and Cu^2+^ signals in ceruloplasmin and free radicals decreased after 10 days and increased after 21 days of therapy [95]. Lastly, Egyptian propolis hydrogel CNF/PVA aids in wound healing by increasing cell viability, adhesion, and spreading [124].

Finally, based on the research findings, the use of nanopropolis shows promising potential for wound healing, supported by its synergistic role in antibacterial, anti-inflammatory, antioxidant, and other activities.

## Challenge and future perspective of nanopropolis for oral drug administration

In this review, the utilization of propolis extract in its nano form or as part of a formulated product has demonstrated a range of beneficial clinical effects in in vitro studies, animal experiments, and human models. Nanopropolis formulations offer several advantages over traditional propolis preparations, including enhanced bioavailability, antimicrobial activity, and tissue penetration. These findings suggest the potential of nano-propolis as an oral medication for addressing various health conditions. However, it is crucial to acknowledge that propolis has been associated with several side effects, including dermatitis, oral mucosal ulceration, laryngeal edema, and, in severe cases, anaphylactic shock. Furthermore, the mechanism of action of propolis, especially in its nano form, remains poorly understood. Therefore, further research must be conducted to comprehensively assess the safety and efficacy of nanopropolis. These aspects should take precedence in future studies, guiding the development and optimization of nano-propolis formulations for potential therapeutic use.

## Conclusions

Propolis nanoformulations are proven to have extraordinary biological potential for several disease challenges, while the challenges faced in developing nanoformulations on a large scale to commercialize them require improvements such as nanocarrier synthesis, standardized evaluation methodology in the framework of strategy process improvement of good manufacturing practices. The current focus of clinical trials primarily revolves around the evaluation of propolis nanoformulations in the post-market phase.
